# Indels in SARS-CoV-2 occur at template-switching hotspots

**DOI:** 10.1186/s13040-021-00251-0

**Published:** 2021-03-20

**Authors:** Brianna Sierra Chrisman, Kelley Paskov, Nate. Stockham, Kevin Tabatabaei, Jae-Yoon Jung, Peter Washington, Maya Varma, Min Woo Sun, Sepideh Maleki, Dennis P. Wall

**Affiliations:** 1 Department of Bioengineering, Stanford University, Stanford, USA; 2 Department of Biomedical Data Science, Stanford University, Stanford, USA; 3 Department of Neuroscience, Stanford University, Stanford, USA; 4 Faculty of Health Sciences, McMaster University, Hamilton, Canada; 5 Department of Computer Science, Stanford University, Stanford, USA; 6 Department of Computer Science, University of Texas Austin, Austin, USA; 7 Department of Pediatrics (Systems Medicine), Stanford University, Stanford, USA

**Keywords:** RNA virus, Recombination, SARS-CoV-2, Genomics

## Abstract

The evolutionary dynamics of SARS-CoV-2 have been carefully monitored since the COVID-19 pandemic began in December 2019. However, analysis has focused primarily on single nucleotide polymorphisms and largely ignored the role of insertions and deletions (indels) as well as recombination in SARS-CoV-2 evolution. Using sequences from the GISAID database, we catalogue over 100 insertions and deletions in the SARS-CoV-2 consensus sequences. We hypothesize that these indels are artifacts of recombination events between SARS-CoV-2 replicates whereby RNA-dependent RNA polymerase (RdRp) re-associates with a homologous template at a different loci (“imperfect homologous recombination”). We provide several independent pieces of evidence that suggest this. (1) The indels from the GISAID consensus sequences are clustered at specific regions of the genome. (2) These regions are also enriched for 5’ and 3’ breakpoints in the transcription regulatory site (TRS) independent transcriptome, presumably sites of RNA-dependent RNA polymerase (RdRp) template-switching. (3) Within raw reads, these indel hotspots have cases of both high intra-host heterogeneity and intra-host homogeneity, suggesting that these indels are both consequences of de novo recombination events within a host and artifacts of previous recombination. We briefly analyze the indels in the context of RNA secondary structure, noting that indels preferentially occur in “arms” and loop structures of the predicted folded RNA, suggesting that secondary structure may be a mechanism for TRS-independent template-switching in SARS-CoV-2 or other coronaviruses. These insights into the relationship between structural variation and recombination in SARS-CoV-2 can improve our reconstructions of the SARS-CoV-2 evolutionary history as well as our understanding of the process of RdRp template-switching in RNA viruses.

**Supplementary Information:**

The online version contains supplementary material available at (10.1186/s13040-021-00251-0).

## Introduction

Researchers around the world are closely monitoring the evolutionary dynamics of SARS-CoV-2 (Severe acute respiratory syndrome coronavirus 2), the virus that causes COVID-19 (coronavirus disease 2019) and the source of the 2020 global pandemic. By studying the mutational patterns of viruses, we can better understand the selective pressures on different regions of the genome, robustness of a vaccine to future strains of a virus, and geographic dynamics of transmission.

Most evolutionary analysis begins with constructing a phylogenetic tree based on observed mutations or variants in lineages of SARS-CoV-2. Most of these mutations are filtered to only single nucleotide polymorphisms (SNPs), as structural variants, particularly deletions, may be sequencing artifacts of low-quality reads or low-coverage genomic regions. This particular pipeline of analysis has two shortcomings. First, it ignores insertions and deletions, despite their known role in viral evolution [1] and the importance of considering all types of mutations when building an accurate phylogenetic tree [2]. Secondly, these phylogenetic trees are typically non-recurrent and do not take into account the possibility of recombination between viral lineages. Parallel research has been done to determine whether or not SARS-CoV-2 lineages have already recombined; however, the conclusions have been mixed [3–5]. Not only does the relatively small number of mutations in the SARS-CoV-2 evolutionary history make it difficult to identify a clearly recombined lineage, additionally, the lack of publicly available raw reads makes it difficult to determine if seemingly recurrent mutations are due to recombination, site-specific hypermutability, or systematic sequencing error.

Recombination plays an integral role in the evolution of RNA viruses, including those implicated in recent epidemics: Comparative genomics studies suggest that SARS as well as a SARS-like coronavirus in bats have recombinant origins [6, 7], co-circulating and recombinant lineages of MERS-CoV were found in dromedary camels [8] and several studies hypothesize SARS-CoV-2 has a recombinant origin from bat coronaviruses, pangolin coronaviruses, or both [9–12].

**Fig. 1:**
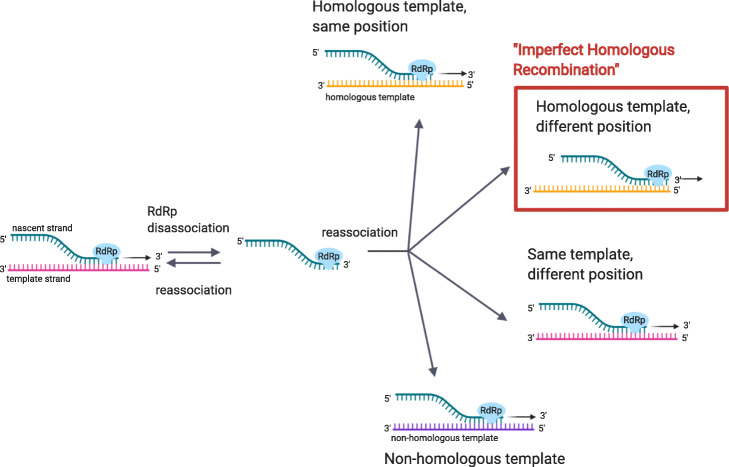
Copy-choice recombination is the presumed primary recombination mechanism for RNA viruses. During negative strand synthesis, the replication complex and the nascent strand disassociate from the template strand. From there, the replication complex can template-switch, or reassociate with a homologous or replicate template strand

It is generally accepted that recombination in RNA viruses is via a copy-choice mechanism by which an RdRp switches template strands during negative strand synthesis, the first step of both sub-genomic transcription and full-genome replication in +ssRNA viruses [13–16]. In this process (Fig. 1), RdRp disassociates from the template strand during synthesis of the nascent strand. From there, several reassociation events can occur. The RdRp can reassociate back to the same template strand, either at the same or a different loci; reassociate with a homologous template, again either at the same or different loci; or reassociate with a non-homologous template. Note that throughout this paper we refer the process of the RdRp reassociating with a homologous template at a different loci as “imperfect homologous recombination.” If the RdRp fails to reassociate, negative strand synthesis will terminate. Outside of known transcription regulatory sites, what causes RdRp to disassociate and reassociate to a different template strand mid-transcription or replication is not well understood [17]. An early study suggested that in the absence of natural selection, RNA virus recombination occurs entirely at random with respect to genome position [18], and is independent of RNA secondary structure or sequence. Successive studies have found secondary RNA structure motifs that lead to RdRp disassociation and subsequent recombination in RNA viruses [19–22].

**Fig. 2:**
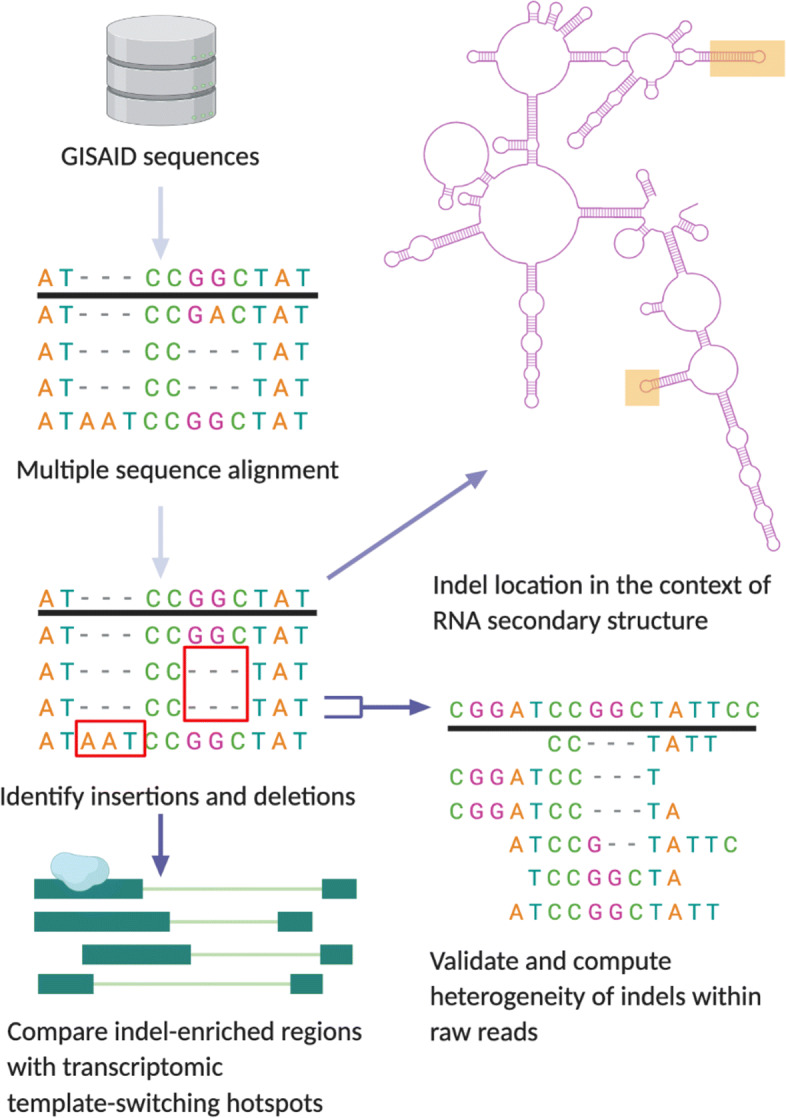
General pipeline of project: using GISAID sequences, we identified indels present in SARS-CoV-2 lineages. We compared the location of these indels to regions of discontinuous transcription breakpoints, computed the heterogeneity of indels using raw reads, and analyzed the indel locations with respect to the secondary RNA structure using a simulation of the folded SARS-CoV-2 RNA molecule

Using 16,662 GISAID sequences [23], we characterize over 100 deletions and insertions in the evolutionary history of SARS-Cov-2 as of early June 2020, and hypothesize that these indels are the result of imperfect homologous recombination. We offer several pieces of evidence that suggest this (Fig. 2). (1) We show that the indels in the consensus GISAID sequences are found in clusters across the genome. (2) Using long-read transcriptomic data [24], we show that these clusters correspond to regions of the genome that have high rates of TRS-independent polymerase jumping, hypothetically RdRp template-switching hotspots. (3) We show that many of these indel hotspots show high rates of heterogeneity in the raw reads, suggesting that even sequences where the consensus sequence does not contain the indel may be undergoing de novo recombination at these sites. We also briefly note that many of these indel clusters are found on “arms” and loop structures within the predicted RNA secondary structure of SARS-CoV-2, suggesting that global RNA secondary structure may play a role in RdRp template-switching in SARS-CoV-2 and other coronaviruses.

## Materials and methods

### Data access and preparation

To obtain the SARS-CoV-2 consensus sequences, we accessed the GISAID sequences on June 3, 2020. We filtered to high-coverage full length (>29kb) sequences, where less than 20 bases were missing, totalling 16,662 sequences.

To obtain the raw reads, we accessed the NCBI SRA run browser on June 3, 2020. We found the accession numbers that corresponded SARS-CoV-2 reads that were full length, short reads from Illumina sequencing machines, and consisted of less than 1 billion total base calls (to speed up computation). We downloaded these using the NCBI’s fastq-dump API.

To compare the regions with enriched numbers of indels to the hypothesized SARS-CoV-2 template-switching hotspots, we used the deep sequencing long-read SARS-CoV-2 transcriptome data published by Kim et al. [24]. We used the reads from the Vero-infected cells, filtered to reads that aligned to the SARS-CoV-2 genome (VeroInf24h.viral_genome.bam).

### Identifying indels

We used MAFFT10 [25] to perform multiple sequence alignment of the GISAID sequences with NC_045512.2 as the reference sequence. We locally realigned indel calls that were synonymous using custom python code, which called indels synonymous if the unions of their flanking regions were the same. (For example, the indels corresponding to AGGCTG-GGT and AGGCTGG-GT would be considered synonymous). We catalogued indels present between positions 100 and 29,800 in the genome, discarding the more error-prone ends of the genome. The subset of indels that were present in 2 or more sequences is shown in (Table 1), and the entire set of indels found among these sequences is shown in Table S1.

**Table 1:** Table of indels (Deletion - D, insertion - I) found in at least two sequences

Start Pos	Length	Type	# Seqs	Countries
222	1	D	2	USA, England
508	15	D	2	USA
510	9	D	5	USA, France, Scotland, England
515	6	D	18	Belgium, United, USA, Greece, Denmark, Australia, England
515	3	D	6	USA, Australia
518	3	D	4	Spain, USA, Netherlands, Denmark
669	3	D	9	India, USA
686	9	D	55	Sweden, Belgium, USA, Saudi, Canada, Israel, Spain, Portugal,
				Netherlands, Iceland, Denmark, Turkey, France, Australia, England
729	9	D	5	Sichuan, Wuhan
1431	3	D	2	USA, Yunnan
1605	3	D	332	Spain, Portugal, Russia, Latvia, Germany, Northern, Australia,
				England, Belgium, USA, Netherlands, Iceland, Denmark, Chile, Wales,
				Greece, France, Sweden, Taiwan, Finland, Scotland, Pakistan, New
3333	3	D	23	Kazakhstan
6501	3	D	2	England
6506	3	D	2	Iceland
6510	6	D	2	India, Australia
6518	6	D	2	USA
11074	3	I	16	United, Portugal, Switzerland, Taiwan, Jamaica, Scotland, Jordan, Australia
12620	3	D	2	Netherlands
14865	2	D	12	Wuhan
18412	1	D	6	Wuhan
20423	3	D	2	USA, Portugal
20965	1	D	4	Wuhan
21991	3	D	14	Belgium, India, USA, Saudi, Netherlands, Slovenia, Jordan, England
25532	3	D	2	USA, France
26159	2	D	2	USA
26351	6	D	2	India
27701	3	D	2	England
27848	382	D	13	Singapore
27910	345	D	2	Bangladesh
28090	6	D	3	USA, Iceland, Australia
28254	1	D	6	Wuhan
29593	2	I	2	USA
29686	1	I	7	Iceland, Thailand, England
29723	44	D	2	Argentina
29726	1	D	2	England
29756	7	D	4	India, USA, Netherlands, England
29760	5	D	2	USA
29761	2	D	5	USA, Australia
29788	2	D	3	England

To test if the indels were more clustered together than expected by chance, we computed the distance between each unique indel start position and its nearest neighbor indel start position. We computed a simulated null distribution by randomly swapping each indel start location with a different loci between 100-29,800 (the regions which we allowed indels to be found in), and recomputed the distance between each start position and its nearest neighbor distances. We performed this 100,000 times to derive an expected null distribution of nearest neighbor distances [26–28]. We then compared our true distribu- tion of nearest neighbor distances using a Mann-U Whitney test. Note that as most of our data was not necessarily normally distributed, we opted for non-parametric statistical tests throughout our analysis.

### Comparing with template-switching hotspots in the transcriptome

**Fig. 3:**
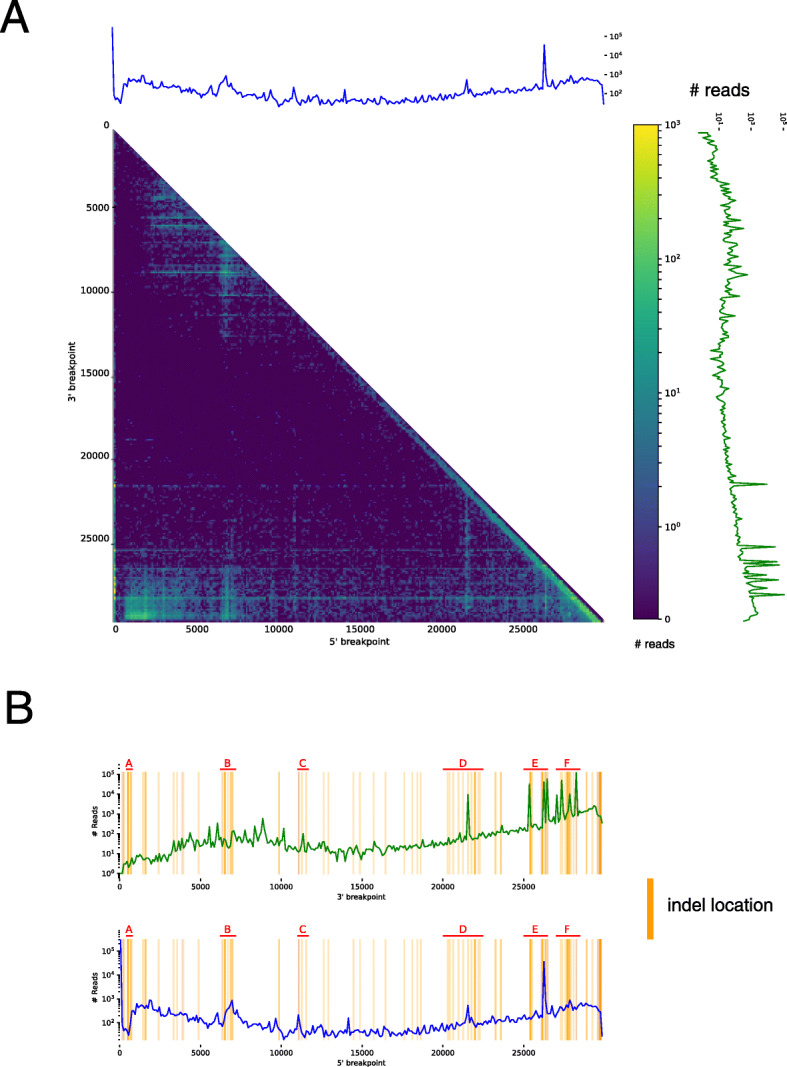
**a** Heatmap of 5’ breakpoint vs 3’ breakpoints from long-read TRS-independent discontinuous transcripts. Reads were considered TRS-independent discontinuous transcripts if the leader sequence was not near the transcription regulatory sequence and the aligned read contained a deletion of 100 or more bases. The heatmap and histograms are in bins of 100 bases. Colors in the heatmap and amplitude of blue and green lines are log-normalized. **b** The log-normalized number of discontinuous transcripts with given 5’ and 3’ breakpoints overlaid with the locations of the indels (by 5’ start position)

Kim *et al* [24] identified non-canonical subgenomic RNAs (sgRNAs) in the SARS-CoV-2 transcriptome characterized by large deletions in the middle of the transcript, presumably a result of the template-switching mechanism for discontinuous transcription. From the viral reads collected in the Kim et al. study, we filtered to reads with a deletion of 100 bases or more relative to the reference genome and computed the locations of the 5’ and 3’ breakpoints. We compared the breakpoint hotspots to the location of the indels we identified in the GISAID sequences (Fig. 3).

To test if the locations of the indels correlated to the locations of the 5’ and 3’ breakpoints in the transcriptome, we created an indel, 5’, breakpoint, and 3’ breakpoint vector each 29,904 (the length of the SARS-CoV-2 genome) elements long to represent the location of indels, the location of the 5’ breakpoints, and the locations of the 3’ breakpoints. From the indels in Table S1, the indel vector consisted of the number of unique deletions at the corresponding loci. The 5’ vector consisted of the number of reads with a 5’ breakpoint at the corresponding loci. The 3’ vector consisted of the number of reads with a 3’ breakpoint at the corresponding loci. We computed the Spearman correlation between the 5’ vector and the indel vector, as well as the 3’ vector and the indel vector. [28,29]

### Indel heterogeneity from raw reads

We analyzed the indels in the context of the raw reads for two major reasons. First, we wished to validate that these indels were in fact true insertions or deletions, and not the result of sequencing error or low-coverage genomic regions. Secondly, we wished to measure intra-host heterogeneity at these sites, to determine whether these indels might be the result of imperfect de novo recombination events (reassociation of RdRp with a homologous template at a different loci, Fig. 1) or located in hypermutable regions, or whether they were inherited from the a viral lineage in the previous host.

We accessed NCBI’s SRA run browser on June 3, 2020 to download the fastq files for the full genomic sequences of SARS-CoV-2. We restricted to Illumina reads, as short reads have smaller error rates than long reads and are less prone to systematic sequencing types of errors [30]. We quality filtered the reads using fastp [31], with a qualified quality phred cutoff of 20, an unqualified percent limit of 20, and a required length of 50. Using NC_045512.2 as the reference, we used bwa-mem [32] to align reads to the reference genome, following the standard paired for single-end read pipelines as appropriate. We marked and removed PCR duplicates using GATK’s MarkDuplicates. We used lofreq to quality score the indels, perform local realignment, and compute indel heterogeneity [33]. We used an in-house python script to visualize the raw read alignment compared to the reference genome for a given sample and indel loci as shown in Fig. 5.

We performed a significance test to see if samples had higher heterogeneity at our catalogued indel sites compared to the rest of the genome. Using the raw reads without an indel as the dominant genotype at a given site (alternate allele frequency as computed by lofreq (AF) ≤.5), we compared the indel frequency at our aforementioned indel sites with the frequency of indels at the rest of the genome. Using a Mann-U Whitney test we computed a *p*-value for the null hypothesis that the heterogenity rates are the same in our indel sites compared to the rest of the genome [28,34,35].

### Predicted RNA secondary structure

It is presumed that RdRp template switching is responsible for the discontinuous transcription and recombination in coronaviruses. While transcription regulatory sites (TRS) govern some of the leader-to-body fusion sites, little is known about what mechanisms are behind TRS-independent transcription and replication. We used RNAfold [36] as well as mxfold [37] with the default parameters, to generate estimates of the secondary structure of the reference SARS-CoV-2 RNA genome. We chose these two folding prediction tools because we wished to use both a thermodynamics-based prediction method, and a machine learning-based prediction method. RNAfold is a commonly used thermodynamics-based prediction method, and mxfold is a recent hybrid (using both machine learning and thermodynamics) prediction method that has been shown to perform well on longer RNA sequences [38].

We used RNApdbee [39,40] and bpRNA [41] to annotate the secondary RNA structures. We visualized all RNA structures using VARNA [42].

To test if indels preferentially occurred in certain secondary structures, we compared the distribution of secondary structures at our indel locations against the distribution of secondary structures in the full genome, using a chi-square test. We computed the distribution of secondary structures by mapping the start locations of each unique indel, and compared that to the distribution of secondary structures in loci 100-29800 (the range which we allowed indels) using a chi-square test. [43] We performed this for every combination of RNA folding prediction software (RNAfold, mxfold) and RNA secondary structure annotation software (RNApdbee, bpRNA).

## Results

### SARS-CoV-2 lineages contain over 100 indels

Ignoring the error-prone and low-coverage 5’ and 3’ ends of the genome, we found 122 total indels between loci 100 and 29,800 (Table S1).

Table 1 shows the most common indels, that is those that were found in two or more sequences. Of these 39 common indels, 24 are deletions or insertions of multiples of 3 bases, and would not result in a frameshift. Most (8) of the indels that would result in a frameshift occur downstream from loci 29500, after the stop codon of the last canonical open reading frame.

Visually, the indel sites appear to be clustered together. To test if this clustering was significant, we computed the distance between each indel start location and the nearest indel. We compared this distribution to a simulated null distribution and show that the observed indels are closer together to each other than expected by chance (Mann-U Whitney *p*-value 1.7x10^−15^).

### Indels cluster at SARS-CoV-2 template-switching hotspots

The coronavirus transcriptome is characterized by discontinuous transcription events. During discontinuous transcription, RNA-dependent RNA polymerase (RdRp) ‘jumps’ from a 5’ breakpoint to a 3’ breakpoint. This discontinuous transcription may occur across a single genome of a virus or it may involve 2 copies of the RNA genome, with the RdRp switching from one template (leader) to another (body) mid-transcription. [13,16,44] According to the prevailing model, leader 5’ breakpoints and body 3’ breakpoints occur at short motifs called transcription-regulatory sequences (TRSs) adjacent to open reading frames [45,46]. In a deep sequencing study of the SARS-CoV-2 transcriptome, Kim et. al. found that there were many discontinuous transcription events not characterized by TRS (known as TRS-L-independent fusion), with both the 5’ and 3’ breakpoints clustered at specific regions of the genome. The mechanism behind TRS-L-independent fusion is not currently well understood.

We found that the number of unique indels at a given loci and the number of discontinuous transcripts with a 5’ or 3’ breakpoint were highly correlated (Spearman *p*-value 3.5x10^−5^ for indel count vs 3’ count, p value 5.7x10^−8^ for indel count vs 5’ count). In Fig. 3, we note several regions of interest, where the genome was enriched for indels identified from the GISAID sequences and where the genome was enriched for either 5’ or 3’ breakpoints.

### Indels have intra-host heterogeneity

**Fig. 4:**
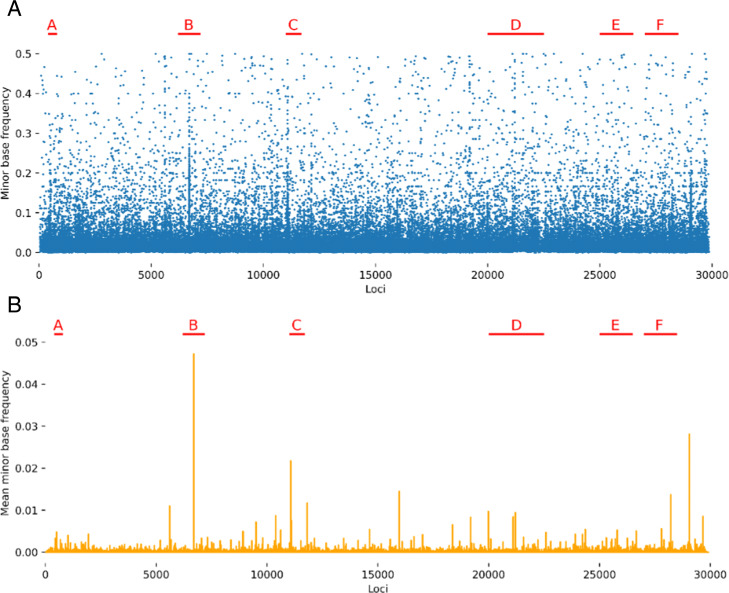
Rates of heterogeneity in raw reads. **a** Alternate allele frequency vs indel location as computed from raw reads by lofreq. **b** Mean alternate allele frequency vs indel location as computed from raw reads by lofreq

Using the raw reads, we found high rates of intra-host heterogeneity for the indels. Figure 4 shows the rates of heterogeneity for indels at each loci as computed from the raw reads. Many of the same regions of the genome enriched for 5’ and 3’ breakpoints in the transcriptome, particularly regions B, C, D, and F, also have high rates of heterogeneity for small deletions and insertions within the raw reads.

**Fig. 5:**
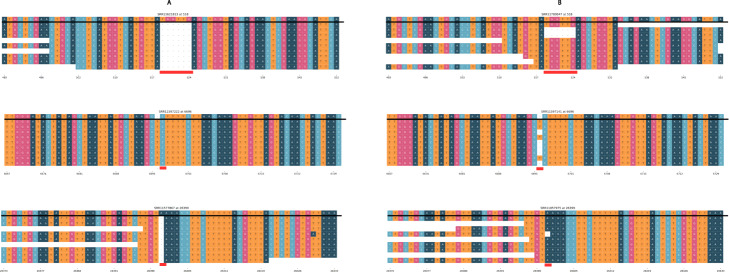
Raw read alignment for several common deletions Examples of the raw reads (10 raw reads randomly chosen) for indels with strong homogeneity in one host and high heterogeneity in another host. The red line the reads below indicates where the insertion or deletion of interest is. A “-” indicates a gap in a read relative to the reference, or vice versa, whereas blank spaces simply indicate the ends of a read. (**a**) Examples of raw reads with a strong homogeneity at the indel, suggesting that the indel arose from an event in a previous host. (**b**) Examples of raw reads with intra-host heterogeneity at the indel, suggesting that these indels are de novo events

As seen in Fig. 5, many indels have samples with high heterogeneity. From the raw reads without an indel as the dominant genotype at a given site, we compared the indel frequency at our aforementioned indel sites, with the alternative variant frequency of indels at the rest of the genome. We show that our indel sites have higher rates of heterogeneity (Mann-U Whitney *p*-value 0.0005) compared to the rest of the sites in the genome.

However, we also see samples with high homogeneity for a given structural variant call, as shown in Fig. 5. This suggests that structural variants may occur by either a recombination or mutation event in a previous host (resulting in high intra-host homogeneity), or from de novo recombination within a current host (resulting in high intra-host heterogeneity).

### Indels cluster at arms and loops in the secondary RNA structure

**Fig. 6:**
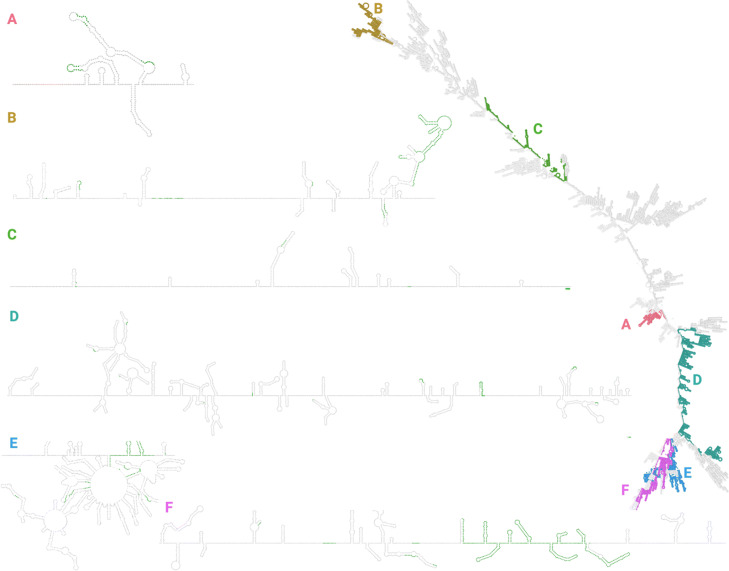
RNA structure was simulated using RNAfold and visualized with VARNA. The zoomed-in subsections of RNA are from the selected regions of both high indel and discontinuous transcription breakpoint enrichment. Green areas represent regions with an indel. Note that the structures zoomed-in subgenomic RNAs have been manually refined to avoid overlap of loops for easier visualization

**Fig. 7:**
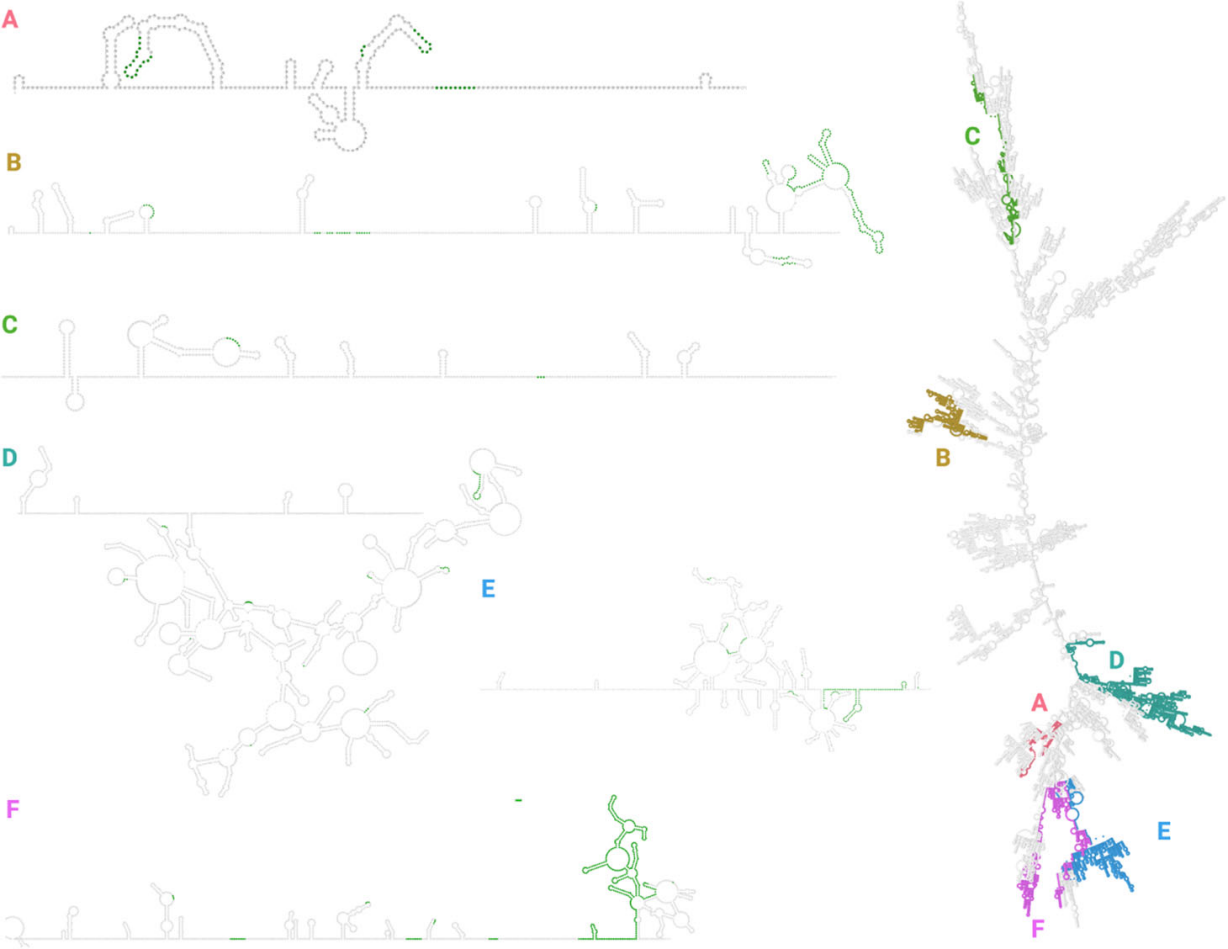
RNA structure was simulated using mxfold and visualized with VARNA. As in Fig. 6, the zoomed-in subsections of RNA are from the selected regions of both high indel and discontinuous transcription breakpoint enrichment. Green areas represent regions with an indel

To see if there were any obvious structural motifs associated with indels or hypothesized recombination hotspots, we simulated the secondary RNA structure of SARS-CoV-2 and analyzed the locations of the indel clusters, using both RNAfold (Fig. 6 and Fig. S1) and mxfold (Fig. 7 and Fig. S2). From Figs. 6 and 7, RNAfold and mxfold both predict indel clusters to be on “arms” of the folded RNA; that is, highly accessible regions that are extended away from the RNA backbone. In particular, regions B, and D-F are consistently located on the some of the furthest extensions of the folded RNA molecule.

We annotated the RNA structures using both RNApdbee [39,40] and bpRNA [41], which derive secondary structures. RNApdbee can annotate stems, loops, and single strands, while bpRNA can gives slightly more sophisticated annotations such as stem loops, bulges, and inner loops.

To test if the indels preferentially occurred in any secondary structure, we annotated the RNA structures using RNApdbee [39,40] and bpRNA [41] and compared the distribution of secondary structures at indel sites to the overall distribution of secondary structures.

We found that in every combination of RNA folding prediction software and secondary structure annotation program, indel sites were preferentially were enriched for loop structures and underenriched for stem structures: For the bpRNA annotations, indel starts were disproportionally in hairpin, internal, and multiloops, rather than bulges and stems. This was true for both the structure predicted by RNAfold (chi-square *p*-value 6x10^−4^) and the structure predicted by mxfold (chi-square *p*-value 6x10^−6^). For the RNApdbee annotations, indel starts were disproportionally in loops and single strands rather than in stems. Again, this pattern held for both RNAfold predictions (chi-square *p*-value 1x10^−4^) and mxfold predictions (chi-square *p*-value 4x10^−4^).

## Discussion

We have catalogued over 100 indels in the SARS-CoV-2 genome, a type of mutation that was largely ignored in the early analysis of SARS-CoV-2 evolutionary history. Via the GISAID consensus sequences, publicly available raw reads, long-read deep transcriptomic data, and simulated RNA structure, we show several independent pieces of evidence that suggest that these indels are artifacts of recombination, and that SARS-CoV-2 contains several recombination hotspots.

Interestingly, using sequence-based recombination detection approaches, previous studies have identified several of our hypothesized recombination hotspots as recombination breakpoints in SARS-CoV-2 and other related coronaviruses. Lau et al. found evidence of the N and ORF8 proteins of SARS being acquired from recombination between horseshoe bat viruses. They identified recombination breakpoints at 20900, 26100, 27128, and 28635 [6] - which correspond well to our indel hotspots D, E, and F. Hom et al. also identified a possible recombination breakpoint around 21495 in tracing SARS from a bat coronavirus [7], corresponding to indel-enriched region D. Lam et al. identified a possible recombination schema for SARS-CoV-2 from Malayan panglolin viruses and bat CoVs with breakpoints around 11000, 21000, 23000, 24000 [10], which corresponds to C, D, and E indel-enriched regions. Analysis on the sarbecovirus recombinant origins of SARS-CoV-2 identified possible recombination breakpoints at 1684, 3046, 9237, 11885, 21753, 22773 and 24628. [47]. 1684 is close to 1605, at which 332 of the GISAID sequences we analyzed have a deletion 3 bases long. The latter 4 breakpoints fall close to or within our identified indel-enriched regions C, D, and E. We also see several deletions between 2500-3500 (possibly linked to the breakpoint hotspot at 3046), though we see no indels within 500b of 9237.

Globally, regions enriched for indels and transcriptional breakpoints appear to fall on “arms” of the simulated folded RNA molecule. We hypothesize the because these regions of the RNA molecule are extensions from the backbone, they are easily accessible and therefore the RdRp can “jump” between homologously aligned replicate molecules. We note that this is a crude representation of the secondary RNA structure; it ignores the interactions between genome and nucleocapsid, uses only the reference sequence and does not capture how mutations might change the folded RNA structure in different lineages, ignores psueodknots, and only shows the primary consensus fold. Furthermore, RNA folding prediction algorithms have historically decreasing performance on longer RNA molecules [38]. However, given that both RNAfold and mxfold both showed indels significantly enriched in loop structures, it seems possible that conserved RNA structure does play some role in RdRp disassociation. Additional work needs to be done to determine if additional local sequence or structural motifs exist that guide RdRp disassociation.

There are several alternative explanations for these highly enriched regions of indels, but we believe that they are unsupported by the combined evidence in the GISAID sequences, raw reads, and transcriptome data. First of all, addressing the obvious possibility of systematic sequencing or alignment error, we see no signs in the raw read data that the indels are due to such error types. The indels occur in many Illumina samples, which are not prone to systematic sequencing errors, and many samples have nearly 100% homogeneous calls for a given indel. Many heterogeneous reads have alternate variant frequencies too high to be consistent with Illumina error profiles [48, 49].

Another theory is that perhaps indels occur at hypermutable sites within the genome, and it is either by chance that they appear to be clustered in several regions, or selective pressure weeds out indels in other areas of the genome. However, recall that these regions are also enriched for 5’ and 3’ breakpoints in the transcriptome, which we calculated by only considering reads with a deletion >100 bases. If these sites are in fact hypermutable, then they are also hypermutable for larger indels as well; selective pressure would not be acting on the transcriptome in such a manner. It seems possible, however, that there might be additional template-switching hotspots that can be seen in the discontinuous transcriptome, but not in the regions of indel enrichment because selective pressure makes SARS-CoV-2 unable to handle indels in this region. [50] For example, there seems to be enrichment of 5’ end breakpoints in the discontinuous transcriptome between loci 8000 and 9000, however we found no indels in that region (see Fig. 3); perhaps a indel in this region would result in a dysfunctional phenotype.

Finally, these indels might be the result of RdRp disassociating and reassociating from one location to another on the same strand of RNA, rather than from a template strand to a nascent strand of a viral replicate. This would mean that these indels are not created from template switching between two separate viral strands, but from RdRp disassociating and reassociating on the same viral strand. This is possible; however it is likely that if an area is a hotspot for RdRp jumping within the same strand, it is consequently a hotspot for RdRp template switching between two different template strands. Recombination between two or more SARS-CoV-2 template strands could be verified experimentally by measuring recombination rates between mutant viral lineages, or computationally by finding a patient that has been co-infected by two different SARS-CoV-2 lineages with discernible mutations on either side of a recombination breakpoint. This computational verification may be difficult as it would require co-infection in a patient, the presence of both lineages within the same cell, recombination, and the recombinant lineage to make it into the sequencing reads.

We emphasize how valuable the raw or aligned reads are for better understanding of SARS-CoV-2 evolutionary dynamics. Although the consensus sequences such as those on GISAID provide some information about mutational patterns and evolutionary dynamics, there are several shortcomings in consensus sequences that raw reads can address. As we have shown, using raw reads we can quantify site-specific mutability. An estimate of per-site variation, both for SNPs and for indels, is essential for building accurate phylogenetic trees [51, 52], which can then be used to trace the spread of SARS-CoV-2 and identify recurrent mutations or sites under high selective pressure [53]. Furthermore, as SARS-CoV-2 continues to spread and inevitably recombines with either itself in the form of a different lineage, another coronavirus, or another RNA molecule, the raw reads with provide a clearer understanding of recombination patterns than consensus sequencing can.

We therefore urge the scientific community to make their raw reads publicly available if possible. While there are possible privacy concerns with human DNA or RNA contamination in the data, most pipelines that generate a consensus sequence involve filtering our reads aligning to the human genome, thereby maintaining privacy and lowering barriers for open access to the scientific community.

In conclusion, we have catalogued over 100 indels present in the SARS-CoV-2 evolutionary history thus far and shown several independent pieces of evidence that these clusters of indels indicate recombination hotspots. An improved understanding of structural variation as well as recombination in coronaviruses will improve phylogenetic reconstructions of the evolutionary history of SARS-CoV-2 and other coronaviruses, and is one step closer to understanding the outstanding questions surrounding the RdRp template-switching mechanism in RNA viruses.

## Supplementary Information

**Additional file 1** **Table S1** containing descriptions of all indels between loci 100-29800 as detected in the GISAID sequences, **Figures S1** and **S2** showing a locations of all indels in the context of RNAfold and mxfold, respectively.

## Data Availability

All genomic sequencing data is publicly available on NCBI, and the transcriptomic data was previously published by Kim et al. [24]. Source code and analysis is freely available at github.com/briannachrisman/sars-cov2-SVs.

## Abbreviations

COVID-19: Coronavirus disease 2019
SARS-CoV-2: Severe acute respiratory syndrome coronavirus 2
Indel: Insertion or deletion
TRS: Transcription regulatory site
RdRp: RNA-dependent RNA polymerase
sgRNAs: Sub-genomic RNAs
