# Supplementary material for: Indels in SARS-CoV-2 occur at template-switching hotspots
Source: BioData Min. 2021 Mar 20;14:20. doi: 10.1186/s13040-021-00251-0 (PMC7980745; doi:10.1186/s13040-021-00251-0)
Supplement: Supplementary file 1 — Additional file 1 Table S1 containing descriptions of all indels between loci 100-29800 as detected in the GISAID sequences, Figures S1 and S2 showing a locations of all indels in the context of RNAfold and mxfold, respectively. [file 13040_2021_251_MOESM1_ESM.pdf]

1 Supplementary Table S1

Start Pos	Length	Type	# Sequences	Countries
130	1	I	1	England
203	1	I	1	Bangladesh
222	1	D	2	USA, England
263	1	D	1	USA
508	15	D	2	USA
510	9	D	5	USA, France, Scotland, England
514	9	D	1	Australia
515	6	D	18	Belgium, United, USA, Greece, Denmark, Australia, England
515	3	D	6	USA, Australia
518	3	D	4	Spain, USA, Netherlands, Denmark
519	6	D	1	England
669	3	D	9	India, USA
686	9	D	55	Sweden, Belgium, USA, Saudi, Canada, Israel, Spain, Portugal, Netherlands, Iceland, Denmark, Turkey, France, Australia, England
729	9	D	5	Sichuan, Wuhan
1431	3	D	2	USA, Yunnan
1577	1	D	1	Wuhan
1598	6	D	1	England

1605	3	D	332	Spain, Portugal, Russia, Latvia, Germany, Northern, Australia, England, Belgium, USA, Netherlands, Iceland, Denmark, Chile, Wales, Greece, France, Sweden, Taiwan, Finland, Scotland, Pakistan, New
2402	27	D	1	Spain
3333	3	D	23	Kazakhstan
3547	9	D	1	Australia
3852	1	I	1	Spain
3933	9	D	1	USA
4879	3	D	1	USA
6311	1	I	1	India
6374	9	D	1	Australia
6501	3	D	2	England
6506	3	D	2	Iceland
6510	6	D	2	India, Australia
6518	6	D	2	USA
6684	3	D	1	Australia
6853	6	D	1	India
6876	7	D	1	Spain
6965	9	D	1	Australia
6996	146	D	1	Spain
9856	3	D	1	Denmark
9858	3	D	1	England
11071	3	I	1	England
11074	3	I	16	United, Portugal, Switzerland, Taiwan, Jamaica, Scotland, Jordan, Australia
11075	1	D	1	New
11271	6	D	1	Iceland
11541	3	D	1	USA
12620	3	D	2	Netherlands
12910	2	D	1	Wuhan
14463	2	D	1	Wuhan
14865	2	D	12	Wuhan

15714	1	D	1	Wuhan
16445	1	D	1	Wuhan
17609	3	D	1	Wuhan
18109	1	D	1	Wuhan
18412	1	D	6	Wuhan
18628	2	D	1	Wuhan
20298	3	D	1	USA
20423	3	D	2	USA, Portugal
20636	1	D	1	Wuhan
20965	1	D	4	Wuhan
21248	3	D	1	Spain
21562	1	D	1	USA
21765	6	D	1	England
21975	21	D	1	USA
21980	3	D	1	Sichuan
21982	12	D	1	England
21991	3	D	14	Belgium, India, USA, Saudi, Netherlands, Slovenia, Jordan, England
22194	3	D	1	England
22289	6	D	1	Sichuan
23228	3	D	1	Netherlands
23286	1	D	1	Wuhan
23585	15	D	1	Malaysia
23595	30	D	1	Hong
25394	6	D	1	Wuhan
25416	9	D	1	USA
25417	6	D	1	Scotland
25432	6	D	1	Scotland
25432	4	D	1	Portugal
25532	3	D	2	USA, France
26078	3	I	1	USA
26155	3	D	1	USA
26158	4	D	1	India
26159	2	D	2	USA
26159	3	D	1	USA
26161	1	D	1	Wuhan
26338	6	D	1	India
26351	6	D	2	India
26358	109	D	1	Spain
26465	3	D	1	Spain

26494	1	I	1	USA
27294	3	D	1	Australia
27387	10	D	1	Israel
27574	3	D	1	USA
27694	4	D	1	Thailand
27695	6	D	1	Vietnam
27698	6	D	1	Belgium
27699	3	D	1	Japan
27701	3	D	2	England
27721	3	D	1	England
27792	3	D	1	Kazakhstan
27792	2	D	1	Vietnam
27795	3	D	1	USA
27848	382	D	13	Singapore
27910	345	D	2	Bangladesh
28090	6	D	3	USA, Iceland, Australia
28254	1	D	6	Wuhan
28271	1	I	1	Oman
28895	9	D	1	Scotland
28899	9	D	1	England
29251	1	D	1	Wuhan
29567	2	D	1	Japan
29593	2	I	2	USA
29683	1	D	1	Chile
29686	1	I	7	Iceland, Thailand, England
29705	1	D	1	Israel
29723	44	D	2	Argentina
29726	1	D	2	England
29729	7	D	1	USA
29743	2	I	1	USA
29746	3	D	1	Israel
29756	7	D	4	India, USA, Netherlands, England
29760	5	D	2	USA
29761	2	D	5	USA, Australia
29762	5	D	1	USA
29776	1	I	1	USA
29788	2	D	3	England

Table S1: All indels between loci 100-29800 as detected in the GISAID sequences. ”I” is for insertion, and ”D” for deletion.

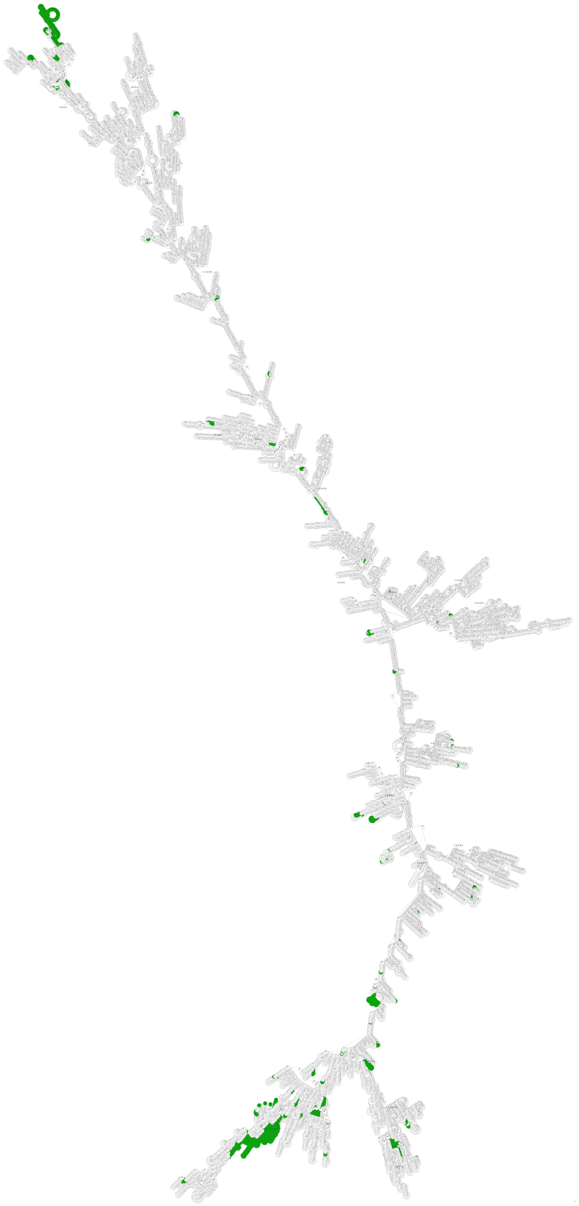

rnafold

Figure S1: RNA secondary structure as predicted by `RNAfold`. Locations of deletions and insertions are colored in green.

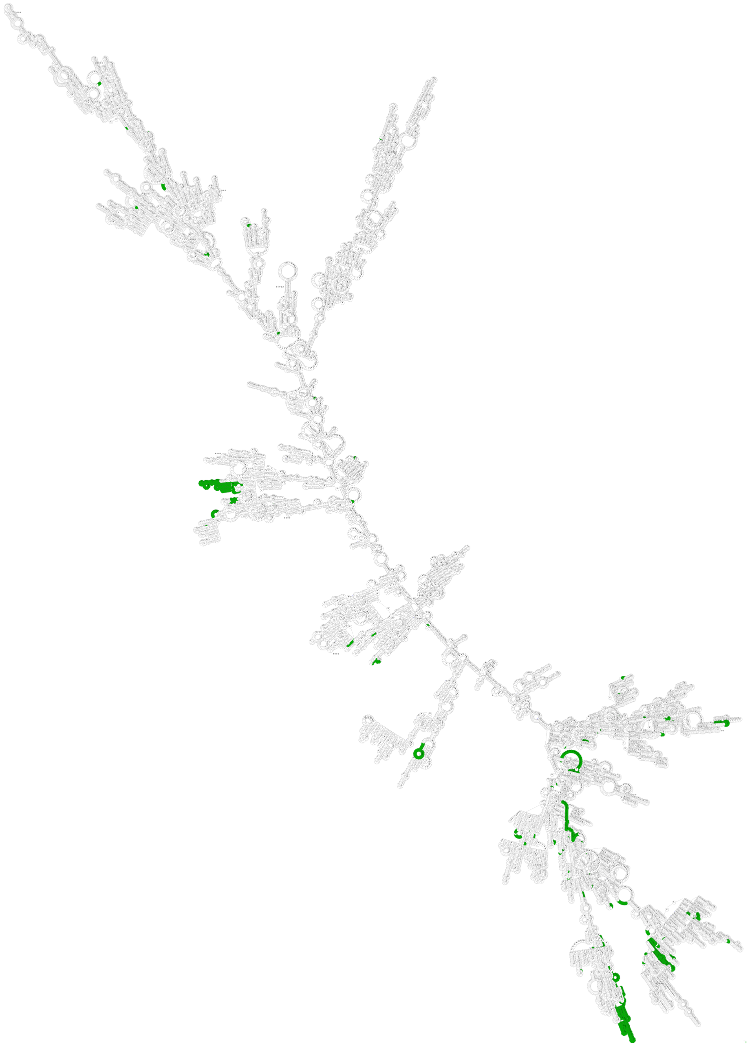

mxfold

Figure S2: RNA secondary structure as predicted by `mxfold`. Locations of deletions and insertions are colored in green.
